# Resource partitioning and amino acid assimilation in a terrestrial geothermal spring

**DOI:** 10.1038/s41396-023-01517-7

**Published:** 2023-09-23

**Authors:** Dengxun Lai, Brian P. Hedlund, Rebecca L. Mau, Jian-Yu Jiao, Junhui Li, Michaela Hayer, Paul Dijkstra, Egbert Schwartz, Wen-Jun Li, Hailiang Dong, Marike Palmer, Jeremy A. Dodsworth, En-Min Zhou, Bruce A. Hungate

**Affiliations:** 1 School of Life Sciences, University of Nevada Las Vegas, Las Vegas, NV USA; 2 Nevada Institute for Personalized Medicine, University of Nevada Las Vegas, Las Vegas, NV USA; 3 Center for Ecosystem Science and Society, Northern Arizona University and Department of Biological Sciences, Northern Arizona University, Flagstaff, AZ USA; 4 State Key Laboratory of Biocontrol, Guangdong Provincial Key Laboratory of Plant Resources and Southern Marine Science and Engineering Guangdong Laboratory (Zhuhai), School of Life Sciences, Sun Yat-Sen University, Guangzhou, China; 5 State Key Laboratory of Biogeology and Environmental Geology, China University of Geosciences, Beijing, China and Department of Geology and Environmental Earth Science, Miami University, Oxford, OH USA; 6 Department of Biology, California State University, San Bernardino, CA USA; 7 School of Resource Environment and Earth Science, Yunnan University, Kunming, China

**Keywords:** Microbial ecology, Water microbiology

## Abstract

High-temperature geothermal springs host simplified microbial communities; however, the activities of individual microorganisms and their roles in the carbon cycle in nature are not well understood. Here, quantitative stable isotope probing (qSIP) was used to track the assimilation of ^13^C-acetate and ^13^C-aspartate into DNA in 74 °C sediments in Gongxiaoshe Hot Spring, Tengchong, China. This revealed a community-wide preference for aspartate and a tight coupling between aspartate incorporation into DNA and the proliferation of aspartate utilizers during labeling. Both ^13^C incorporation into DNA and changes in the abundance of taxa during incubations indicated strong resource partitioning and a significant phylogenetic signal for aspartate incorporation. Of the active amplicon sequence variants (ASVs) identified by qSIP, most could be matched with genomes from Gongxiaoshe Hot Spring or nearby springs with an average nucleotide similarity of 99.4%. Genomes corresponding to aspartate primary utilizers were smaller, near-universally encoded polar amino acid ABC transporters, and had codon preferences indicative of faster growth rates. The most active ASVs assimilating both substrates were not abundant, suggesting an important role for the rare biosphere in the community response to organic carbon addition. The broad incorporation of aspartate into DNA over acetate by the hot spring community may reflect dynamic cycling of cell lysis products in situ or substrates delivered during monsoon rains and may reflect N limitation.

## Introduction

Dissolved organic carbon (DOC) is an important component of Earth’s carbon cycle. Labile DOC is the most dynamic organic matter pool in any aquatic system and plays an important role in all biogeochemical cycles [1, 2]. In the ocean, heterotrophic microorganisms can quickly assimilate and respire labile DOC [3], and in turn, the growth and activity of planktonic heterotrophs is impacted by the bioavailability and characteristics of labile DOC [4–6]. One study tracked the assimilation of six ^13^C-labeled labile substrates into DNA, including acetate and amino acids, by microbial communities in coastal seawater, showing that microorganisms that assimilate specific organic substrates are phylogenetically related [7]. Such phylogenetic conservation of resource utilization reflects similar distribution patterns among related microorganisms and/or similar lifestyles [8]. Similar results were obtained using 11 different organic substrates, also in coastal seawater [9], and the authors proposed two different resource utilization strategies: generalists and specialists. While carbon source assimilation into DNA was either high or low in specialists, generalists incorporated intermediate levels of most or all substrates. Despite numerous studies investigating the roles of specific microorganisms in labile DOC dynamics in marine and other aquatic systems, very little is known about the utilization of labile DOC in hydrothermal systems that may resemble environments in which life first arose [10–12].

In terrestrial hydrothermal systems, DOC can originate from biological sources including nitrogen-depleted allochthonous organic matter from plants and soils, from autochthonous organic matter from thermophilic autotrophs, or from subsurface abiotic processes such as Fischer-Tropsch synthesis and Sabatier-type reactions that require high temperature and pressure [13–15]. Regardless of the source, both fresh and ancient organic materials are susceptible to thermal alteration and decomposition, which also impact labile DOC composition [16, 17]. The relative contributions of DOC with allochthonous and autochthonous origins differ among hot springs. Nye and colleagues characterized DOC in 222 terrestrial springs, 30 from the Tengchong hydrothermal region in China and 192 from Yellowstone National Park in the USA [18]. DOC concentrations ranged from 16 µM to 3 mM, with allochthonous organic matter (i.e., humic-like component) dominating in weakly acidic and circumneutral springs, and low-molecular-weight organic matter (i.e., protein-like component) interpreted to be of hydrothermal origin in alkaline springs with lower DOC concentrations. Humic-like and protein-like components were the main organic carbon signatures in these springs, in addition to an abundant acid-soluble lignin derivative that was exclusively in acidic springs [18]. Moreover, the amount of allochthonous organic carbon in hot springs can be influenced by seasonal precipitation [19, 20], which in turn affects both the quantity and character of labile DOC pools available for heterotrophic thermophiles.

Although few studies have assessed labile DOC utilization in terrestrial geothermal springs, whole-community studies suggest heterotrophic activity of thermophiles in situ may be underappreciated. One study of acidic and circumneutral springs in Yellowstone National Park described high rates of formate and acetate mineralization and formate-induced suppression of autotrophy, suggesting that facultative autotrophs and mixotrophs favor organic carbon assimilation [21]. A separate study found up to 49-fold increases in the instantaneous rate of oxygen consumption by microbial communities in ~80 °C sediments and spring water amended with organic acids or yeast extract and peptone, providing indirect evidence for the use of different organic carbon pools by native communities [22]. Another study demonstrated mineralization of ^13^C-labeled organic compounds, including glucose, citrate, succinate, pyruvate, acetate, and amino acids, in 65 to 95 °C sediment microcosms [23]. It is worth noting that these studies focused on the bulk community. In contrast, the activities of individual thermophiles in situ have rarely been explored.

One method to identify microorganisms that assimilate specific components of labile DOC is stable-isotope probing (SIP). SIP was developed as a tool in microbial ecology over twenty years ago to identify populations actively assimilating labeled compounds of interest into DNA [24, 25]. However, traditional nucleic acid SIP is not quantitative because the DNA or RNA is only separated into two fractions – “heavy” (active) and “light” (inactive) – and guanine and cytosine content is not accounted for, such that high-GC organisms can erroneously be identified as active and low-GC organisms can be misidentified as inactive. Recently, quantitative stable isotope probing (qSIP) was developed to address these deficiencies [26]. qSIP has subsequently been applied to demonstrate that most taxa are active in wet soils [27], better understand organic matter priming in soils [26, 28], illuminate the interactions between soil minerals and bacteria [29], identify highly active bacterial predators and symbionts [30, 31], and probe autotrophy and DOC assimilation in benthic lacustrine sediments [32]. Yet, to date, qSIP has not yet been applied to probe the functions of microorganisms in extreme environments.

In this study, qSIP was applied to assess microbial activity in ~74 °C, pH 7.3 carbonate sediments in Gongxiaoshe Hot Spring during the winter dry season. The geochemistry of Gongxiaoshe Hot Spring has been reported on several different dates [19, 20, 33]. It is relatively stable, with source pool pH and temperatures ranging from pH 7.29-7.7 and 73.8-75 °C. The dissolved oxygen concentration is low (1.5 mg/L), as are dissolved organic carbon (DOC ≤ 1.5 mg/L) and nitrogen (NH_4_^+^/NH_3_ ≤ 0.1 mg/L; NO_2_^-^/NO_3_^-^ ≤ 0.1 mg/L; total N ≤ 0.4 mg/L). The sediments also have low total organic carbon (TOC ≤ 11.6 mg/g) and very little total organic nitrogen (TON ≤ 0.1 mg/g).

Gongxiaoshe Hot Spring is one of many geothermal springs in the Indo-Burma Range of southwest China that are situated in a subtropical climate and are thought to be driven by latent heat from volcanic activity during the Pliocene and Miocene [34]. Springs in this region are exposed to large influxes of terrestrial organic carbon during the summer monsoon season [19]. This increase in DOC during the monsoon season is accompanied by large increases in soil mesophiles in the springs, suggesting that monsoon rains deliver both terrestrial soil organic matter and microorganisms to the springs through surface runoff and/or shallow recharge [19, 20].

In our experiments, two components of the labile DOC pool – the organic acid acetate and the amino acid aspartate – were used in qSIP experiments to assess the responses of specific thermophiles to pulses of labile DOC. While DOC in geothermal systems is complex and poorly understood, acetate was chosen because it is a key intermediate in the carbon cycle as a product of both primary and secondary fermentations and aerobic processing of complex organic carbon such as plant biomass [35–37] and it is commonly detected in hydrothermal systems [21, 38, 39]. Aspartate was chosen as an intermediate in the degradation of proteinaceous biomass – including microbial necromass made available by cell lysis – and because it is a hub for both catabolic and anabolic pathways, as the key intermediate in the aspartate pathway [40, 41]. Aspartate also contains both C and N atoms and could potentially relieve nitrogen limitation. In addition to qSIP, metagenomics was applied to probe the genetic determinants of carbon source utilization. Our results showed strong resource partitioning, a community-wide preference for aspartate incorporation into DNA over acetate, a community-wide increase in biomass only in aspartate-amended microcosms, and near-universal presence of the polar amino acid ABC transporter in the genomes of species that incorporate ^13^C atoms from aspartate into DNA, hereafter termed “utilizers”.

## Methods

### Sample collection and quantitative stable-isotope probing (qSIP)

**Fig. 1:**
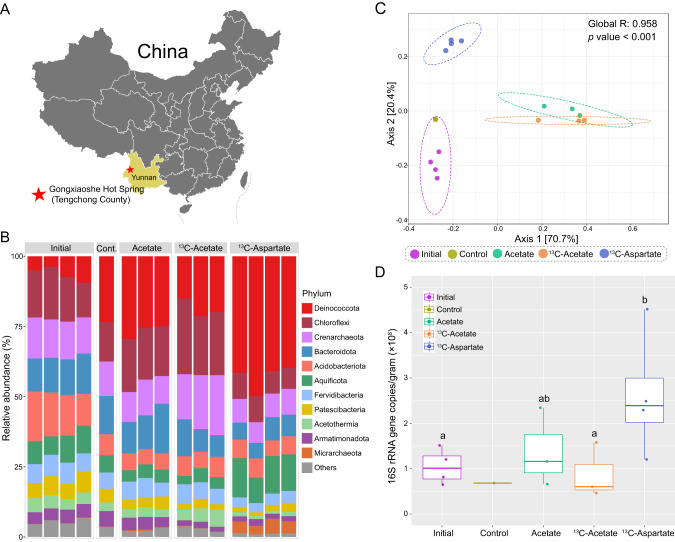
Research location and microbial community response to acetate or aspartate addition. **A** Location of Gongxiaoshe Hot Spring (red star). **B** Bar plots showing the relative abundance of microbial phyla before incubation (initial), after incubation without added substrate (control), or with ^13^C-acetate (*n* = 3) or ^13^C-aspartate (*n* = 4). **C** Effect of incubation on beta diversity. Principal coordinate analysis (PCoA) based on Bray-Curtis dissimilarity index of ASVs followed by ANOSIM significance test. The five groups are: initial (purple), control (brown), natural abundance acetate (green), ^13^C-acetate (orange), and ^13^C-aspartate (blue). Dotted ellipses represent 95% confidence intervals. **D** 16S rRNA gene copies per gram (wet weight) determined by qPCR. Different letters represent significant differences as determined by Kruskal–Wallis test. (*p* < 0.05).

Homogenized carbonate surface sediment (top ~10 cm; ~4 g; 74 °C) and spring water (4 mL) collected from Gongxiaoshe Hot Spring in Tengchong County (Fig. 1A; GPS location N25.44012°; E98.44081°) during the winter dry season (Jan 5, 2016) was supplemented with sterile 95 atom% ^13^C-acetate (*n* = 3, 9.6 µmol C g^-1^ sediment; ~3.2 mM) or 95 atom% ^13^C-aspartate (*n* = 4, 9.6 µmol C g^-1^ sediment; ~1.6 mM) in 50 mL Falcon tubes, covered with aluminum foil, and incubated in the spring for 48 hours. Identical replicates were amended with acetate with natural abundance ^13^C (*n* = 4, 1.6 µmol C g^-1^ sediment) or with no substrate addition (*n* = 1) to increase the number of ASVs with natural C isotope abundance for the AFE calculation. Following incubation, sediments were transferred aseptically to sterile polypropylene tubes, immediately frozen on dry ice, and transported to the lab. DNA was extracted using the Fast DNA Spin Kit for Soil (MP Biomedicals), separated by isopycnic ultracentrifugation on a CsTFA density gradient, fractionated, and used as templates for 16S rRNA gene PCR and Illumina tag sequencing in parallel with a sample of the homogenized, unincubated sediment (*n* = 4). For each sample, 13-15 fractions were collected and each was sequenced. Primers 515 F (5′-GTGYCAGCMGCCGCGGTAA-3′) and 806 R (5′-GGACTACHVGGGTWTCTAAT-3′) were used to target the V4 region of the bacterial and archaeal 16S rRNA gene [42, 43]. Additionally, a broad-coverage quantitative PCR was used to measure the microbial 16S rRNA gene copy number in each fraction as described previously using the same primers [26].

### Bioinformatic and qSIP data analysis

16S rRNA gene data were analyzed using the Quantitative Insights into Microbial Ecology (QIIME2; version 2021.2) pipeline [44]. Three steps were applied to remove contaminants. First, ASVs that have been reported as contaminants due to reagent and laboratory contamination were filtered [45–47]. Second, the R package decontam [48] was used to identify and remove contaminant ASVs that have an inverse relationship with DNA concentration (threshold=0.05). Third, ASVs classified as members of well-characterized families or genera with no thermophiles or hyperthermophiles were removed manually. Isotope incorporation was quantified by converting taxon-specific shifts in DNA density to atom fraction excess (AFE) ^13^C (https://bitbucket.org/QuantitativeSIP/qsip_repo). Chi-square goodness-of-fit test was performed for statistical analysis of resource partitioning. ASV profiles among sample fractions were investigated to find primary utilizers absent from control samples. Since these ASVs had no associated data prior to labeling, they were not detected by the normal qSIP analysis pipeline and therefore do not have AFE calculations (Supplemental Table 1).

### Phylogenetic signal analysis

The SSU database SSURef_Nr99_138.1 was downloaded from the SILVA rRNA database project (https://www.arb-silva.de/) and imported into ARB [49]. SSU rRNA gene sequences identified by qSIP were aligned using the SINA aligner v.1.5.0 [50] with default parameters and added to the SILVA tree, as part of the SSURef_Nr99_138.1 ARB package, using the Parsimony (Quick add marked) function. Only the inserted sequences were retained in the SSU phylogeny. The phylogenetic tree was visualized in iTOL [51]. Global phylogenetic patterns of acetate and aspartate assimilation were analyzed using Moran’s I [52] and Abouheif’s Cmean [53] statistics in the R software, utilizing several packages including ape, phytools, picante, phylobase and Geiger [54]. To quantify the level of phylogenetic autocorrelation throughout the phylogeny, phylogenetic correlogram analysis was performed using the R package phylosig [55].

### Microbial community analysis

Alpha diversity (Chao1 and Shannon) and beta diversity (PCoA) analyses using Bray-Curtis dissimilarities were performed using R package phyloseq version 1.36 [56] and MicrobiomAnalyst [57]. Pielou’s evenness index was calculated according to the formula described previously [58] and R-package ggplot2 version 3.3.3 [59] was used for figure generation. Linear discriminant analysis (LDA) effect size (LEfSe) analysis was performed using the ImageGP online tool (http://www.ehbio.com/ImageGP/) and figures were assembled using Adobe Illustrator (version CS6).

An ASV-to-ASV co-occurrence network based on changes in abundance in the control and experimental treatments (*n* = 15) was compiled using Gephi version 0.9.2 [60] based on Spearman’s rank correlation coefficients. The calculation was based on the absolute ASV abundance, which is regarded as the gold standard in quantitative microbial ecology [61], by converting relative abundance based on qPCR data. ASVs present in >10% of the samples and with connections with rho >0.7 were used to construct the co-occurrence network (*p* < 0.01).

### Metagenomic DNA extraction and sequencing

54 sediment samples were collected from Gongxiaoshe Hot Spring in Tengchong, China, and other springs in Tengchong County, between 2017 and 2019. For each sample, a total of 20 grams of sediment from each sample was used for DNA extraction. The Powersoil DNA Isolation Kit (MoBio) was used for DNA extractions, and the concentration was measured with a Qubit fluorometer (Invitrogen, USA). Approximately 30 Gbp (2 × 150 bp) of metagenomic data for each sample were generated with a HiSeq 4000 System (Illumina) using a 350 bp insert library at Beijing Novogene Bioinformatics Technology Co., Ltd (Beijing, China) for each sediment sample. The raw reads were quality filtered and trimmed as described previously [62]. High-quality reads were individually assembled with metaSPAdes v. 3.9.0 [63] with the following kmers: -k 33, 55, 77, 99, 111. BBMap v. 38.85 [64] was used to map reads to scaffolds and binning was conducted using MetaBAT2 [65] on scaffolds >2.5 kbp.

### Matching ASVs to genomes

16S rRNA genes were extracted from metagenome-assembled genomes (MAGs) obtained from the Gongxiaoshe sediment metagenomes or from available metagenomes from other Tengchong hot springs using metaxa2 version 2.2 [66]. Genomes matching to each ASV were identified using NCBI BLASTn version 2.9.0 (blast.ncbi.nlm.nih.gov), applying a threshold >95% (Supplemental Table 2). The quality and taxonomy of each matched genome was checked with CheckM [67], CheckM2 [68] and GTDB-TK version 2.1.0 [69], respectively. A representative genome for each ASV was chosen based on (i) the origin of the genome based on proximity to Gongxiaoshe (priority: Gongxiaoshe (location of the study) >Jinze Hot Spring (~50 m away) >Rehai Geothermal Field (~65 km away)) [70]; (ii) estimated genome completeness and contamination; and (iii) sequence identity between the two 16S rRNA genes. Given the dynamic nature of the accessory genome, we acknowledge that matching ASVs to MAGs from different dates and different, but nearby, springs may lead to some uncertainty regarding interpretation of the metabolic traits of the ASVs from the qSIP study. The estimated size of genomes was calculated as: Size_est_  =  (Size_obs_ − (Size_obs_ × contamination))/(completeness). A total of 68 ASVs have matched genomes and the majority (60/68) have a nucleotide similarity above 98%. Proteins were predicted using Prodigal version 2.6.3 [71] for each genome.

### Integration of qSIP results with MAGs

The genomes were screened for genomic potential to synthesize transporters for aspartate and acetate. For aspartate, screens were conducted for the dedicated ABC transporter substrate binding proteins GltI/AatJ (K10001) [72] and Peb1A/GlnH (K10039) [73], the general L-amino acids substrate binding protein AapJ/BztA (K09969), as well as the polar amino acids substrate binding protein ABC.PA.S (K02030). In addition, screens were conducted for the aspartate symporters GltP [74], GltT [75], GltTK [76], GltPh [77], and YbeC [78]. Briefly, protein sequences from Uniprot (http://www.uniprot.org/uniprot/) incorporated into the KEGG database (http://www.genome.jp/kegg/) were downloaded for local database construction and homology searches with BLASTP version 2.9.0 (http://blast.ncbi.nlm.nih.gov) were conducted. As for acetate, the presence of the acetate transporter ActP (K14393) [79] was screened.

Functional annotations of genomes were conducted for high-quality genomes (Supplemental Table 2) with eggnog-Mapper version 2.0 [80]. To differentiate primary utilizers of the labeled compounds from those that incorporate labeled metabolic products of other microorganisms (i.e., “cross-feeders”), we examined both AFE values and the presence of homologs of all known membrane transporters for the two substrates. Under this scheme, likely primary utilizers of aspartate were designated based on both positive median AFE values and the presence of annotated transporters and biochemical pathways to utilize aspartate. ASVs with positive AFE values but without annotated transporters and genes for utilizing aspartate were considered likely cross-feeders and treated as non-primary utilizers. For acetate, the same criteria were used except that the acetate transporter was not required, because acetic acid can enter cells without active transport.

To identify possible mechanisms underlying differences in substrate assimilation into DNA, differential abundance analyses were performed with genes and KEGG modules for two groups: primary utilizers and non-primary utilizers using metgenomeSeq version 1.22.0 [81], Mann-Whitney Test, and DESeq2 [82]. The minimal doubling time was estimated using gRodon package in R which uses codon usage patterns of the genomic data [83]. In some cases when the same genome matched to different AFE values, the higher AFE value was selected.

## Results

### DOC addition results in reproducible changes in sediment microbial communities

Based on 16S rRNA gene amplicon analysis, the microbial community in Gongxiaoshe Hot Spring sediments was similar to that reported previously [19, 33], with a high abundance (>10%) of *Deinococcota, Chloroflexi*, *Crenarchaeota*, *Bacteroidota*, *Acidobacteriota*, *Aquificota*, and *Fervidibacteria* (Fig. 1B). Incubations with either acetate or aspartate increased the relative abundance of some phyla, notably *Chloroflexi* and *Crenarchaeota* with acetate, *Aquificota* and *Micrarchaeota* with aspartate, and *Deinococcota* with both acetate and aspartate (Fig. 1B). LEfSe identified classes, orders, and families enriched with acetate or aspartate (Supplemental Fig. 1). At the family level, acetate addition increased the relative abundance of *Caldiarchaeaceae*, *Nitrosocaldaceae*, *Ignisphaeraceae*, *Fervidicoccaceae*, and *Thermaceae*, whereas aspartate addition increased the relative abundance of *Acidobacteriaceae_*subgroup_1, *Aquificaceae*, and *Thermaceae*. Principal coordinate analysis (PCoA) generated at the ASV level based on Bray-Curtis dissimilarity indices revealed distinct clustering of samples (Fig. 1C), demonstrating a reproducible effect of DOC addition on microbial community structure. An apparent “bottle effect” upon incubation was observed, as there was a shift over time in the microbial community of the unamended control.

Aspartate and acetate addition reduced Shannon diversity, but the component drivers were different. Aspartate addition lowered both ASV richness and evenness, whereas acetate addition decreased ASV richness, but increased evenness (Supplemental Fig. 2). The increase in ASV evenness in acetate-amended incubations suggests a reduction in competitive exclusion among microbes. Aspartate addition increased total 16S rRNA gene copies per g of sediment; however, there was no significant change in the acetate-amended incubations (Fig. 1D).

### Aspartate incorporation into DNA is more widespread than acetate incorporation

**Fig. 2:**
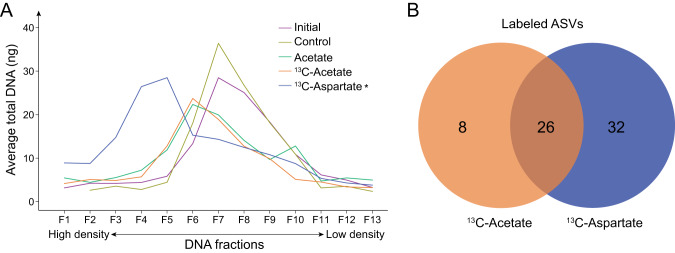
Community-wide preference for aspartate and resource partitioning. **A** Bulk enrichment of ^13^C in DNA extracted from incubations amended with ^13^C-labeled aspartate. The X-axis represents fractions from a cesium chloride density gradient. Significant correlations as determined by Spearman’s rank test are denoted by an asterisk (*p* < 0.05). **B** Venn diagram depicting the number of amplicon sequence variants (ASVs) that assimilated ^13^C-acetate, ^13^C-aspartate, or both, displaying a high degree of resource partitioning (chi-square test of independence, *p* < 0.001).

**Fig. 3:**
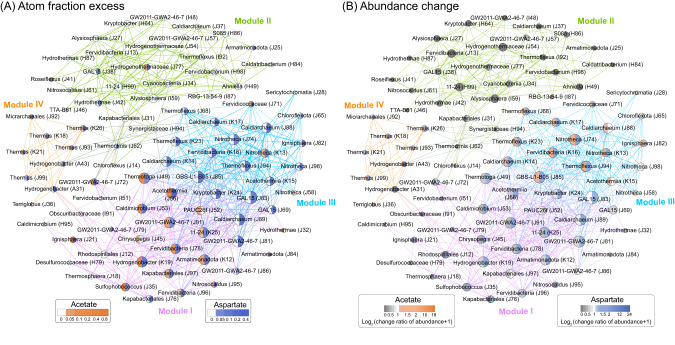
Co-occurrence network analysis based on Spearman correlation. Connections represent strong (Spearman’s rho >0.7) and significant (*p* < 0.01) correlations. Four modules were evident (modularity index >0.415), and each module is distinguished by different line colors. The sizes of nodes are proportional to the number of connections (i.e., number of connections). **A** Nodes are colored based on assimilation of ^13^C from ^13^C-aspartate and ^13^C-acetate (^13^C atom fraction excess), or (**B**) the log2-fold change in absolute abundance. To avoid the logarithm of zeros on the horizontal axis, zero values are indicated as change ratio of abundance +1. The labels show the genus, or the lowest taxonomic rank assigned above genus. The average degree of this network is 7.843, average path length is 2.67, and the clustering coefficient 0.688.

Whereas the abundance of an organism can be affected by a variety of factors, incorporation of the ^13^C label from ^13^C-acetate or ^13^C-aspartate into DNA provides a direct measurement of biosynthesis of newly synthesized DNA. Following the qSIP incubations, the bulk DNA density was highest in the ^13^C-aspartate incubations (Fig. 2A), and incorporation of ^13^C from aspartate was coupled with increases in the absolute abundance of aspartate-utilizing ASVs (Fig. 3; Supplemental Fig. 3). Although there was also an apparent increase in bulk DNA density in the ^13^C-acetate incubations over the initial community DNA, a similar shift occurred in the natural abundance acetate incubations (Fig. 2A), indicating that this increase was driven mostly by a relative enrichment in microorganisms with high GC content rather than incorporation of ^13^C atoms of acetate into DNA. This community preference for aspartate was also supported by the broader utilization of aspartate over acetate among individual ASVs. qSIP analysis showed that 11.8% (58/492) of the ASVs were labeled with ^13^C-aspartate whereas only 6.9% (34/492) were labeled with ^13^C-acetate, and 5.3% (26/492) were labeled with both substrates (Fig. 2B; Supplemental Table 2). The degree of labeling per ASV was not significantly different between ^13^C-aspartate and ^13^C-acetate incubations, but acetate incorporation had a larger range of ASV-specific ^13^C AFE values and was qualitatively less even among ASVs than aspartate incorporation (Supplemental Fig. 4).

In addition to differences in the number of ASVs using ^13^C-aspartate versus ^13^C-acetate, there were also strong differences in the phylogenetic patterns of substrate incorporation into DNA. A strong phylogenetic signal was observed for aspartate utilization (*p* = 0.009 for Moran’s I and *p* = 0.014 for Abouheif’s Cmean). In contrast, there was a weak phylogenetic signal for acetate utilization (*p* = 0.16 for Moran’s I and *p* = 0.08 for Abouheif’s Cmean). The presence of a “phylogenetic gradient”, a decrease of coefficients with increasing phylogenetic distance, was shown for both acetate and aspartate (Supplemental Fig. 5). However, only aspartate utilization showed significant phylogenetic signals across short phylogenetic distances, indicating strong conservation of aspartate utilization at short phylogenetic distances (<0.14).

### Network analysis of primary utilizers and non-primary utilizers

Co-occurrence networks based on the ASVs in the natural samples used to inoculate the qSIP microcosms and the qSIP microcosms themselves were examined to determine whether aspartate and acetate primary utilizers act as coherent metabolic guilds. For this analysis, ASVs that were labeled with a substrate and had genomes that encoded biochemical pathways to incorporate carbon atoms from the substrate into DNA were considered primary utilizers (see Methods). The analysis revealed four distinct modules, with 31 nodes in module I, 28 nodes in module II, 18 nodes in module III, and 9 nodes in module IV (Fig. 3A). Nodes with high degree scores included poorly classified ASVs assigned to GAL15 (33 degrees), GBS-L1-B05 (29 degrees), *Caldiarchaeaceae* (28 degrees), S086 (28 degrees), GW2011-GWA2-46-7 (28 degrees), and *Thermoflexaceae* (27 degrees), suggesting those ASVs are critical for maintenance of the structure and function of the community. The dominant acetate primary utilizers behaved as a defined metabolic guild corresponding mostly to module I (Fig. 3A), but paradoxically, module I and most other ASVs that assimilated acetate decreased in abundance during acetate-amended incubations (Fig. 3B). Instead, most members of module III and some members of module IV increased in abundance in acetate-amended incubations. Aspartate primary utilizers corresponded with modules I, III, and IV (Fig. 3A), consistent with the higher overall labeling of community DNA with ^13^C derived from aspartate, and all three of these modules increased in abundance during incubations with ^13^C-aspartate. Members of module II consisted mostly of ASVs that assimilated neither ^13^C-acetate nor ^13^C-aspartate into DNA and decreased in abundance during the incubations.

### Disproportionate activity of the rare biosphere

**Fig. 4:**
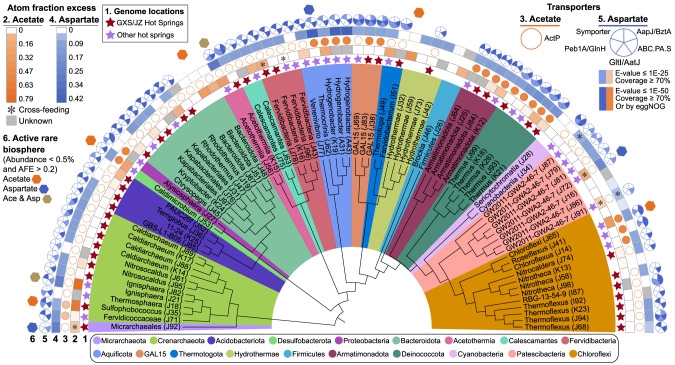
Phylogenetic pattern of genomic potential and ^13^C-acetate and ^13^C-aspartate assimilation. Phylogenetic tree was constructed in ARB software by inserting ASVs into the SILVA reference tree using maximum parsimony based on 16S rRNA gene fragments aligned with the SINA aligner. Only ASVs obtained in both treatments were retained in the tree. Each phylum is colored, with ASV identifiers indicated as genus or the lowest high-confidence rank. ASVs with the best matched genomes (threshold: 95%) are indicated with stars, whose colors indicate the geographical locations of the genomes (red: Gongxiaoshe/Jinze; purple: other hot springs in Tengchong). Solid circles represent annotated transporters for acetate (orange) and aspartate (blue), and the circles are color-coded according to different BLASTP E-value cutoffs. The heatmap and scale indicate the atom fraction excess of ^13^C in DNA after incubation with ^13^C-acetate (orange) or ^13^C-aspartate (blue). Asterisks represent taxa that likely cross-feeding that had positive median AFE values but lacked genomic features for substrate assimilation into DNA (Supplemental Tables 7 and 8). Highly active rare ASVs were shown as hexagons (Supplemental Fig. 3).

All ASVs exhibiting high assimilation of acetate or aspartate (AFE > 0.2) were present at less than 0.5% of the community (Fig. 4; Supplemental Figure 6; Supplemental Table 3). Among them, seven ASVs incorporated acetate and five ASVs incorporated aspartate. Those very active ASVs (AFE > 0.2) belonged to many different phyla, namely *Crenarchaeota* (*Ignisphaera*, acetate and aspartate; *Sulfophobococcus zilligii*, acetate only); *Acetothermia* (*Acetothermiia*, acetate and aspartate); *Aquificota* (*Thermocrinis*, aspartate only); *Bacteroidota* (acetate only); *Chloroflexi* (*Roseiflexus*, acetate and aspartate); *Desulfobacterota* (*Caldimicrobium*, acetate only); *Hydrothermae* (acetate only); *Micrarchaeota* (aspartate only); and *Patescibacteria* (GW2011-GWA2-46-7, aspartate only). Yet, only a few of the very active ASVs became more abundant after incubation (Fig. 3; Supplemental Fig. 3), suggesting ^13^C incorporation into DNA might be uncoupled from cell abundance by viral lysis, parasitism, or predation.

### Genetic ability to incorporate acetate and aspartate into DNA

To test whether the conversion of imported aspartate or acetate into nucleotides is feasible in the taxa that were labeled with ^13^C-acetate or ^13^C-aspartate, and identify genomic markers of substrate assimilation, we matched 73.8% of the ASVs with MAGs from metagenomes derived from Gongxiaoshe Hot Spring (9 MAGs), Jinze Hot Spring (17 MAGs; distance: ~50 m), or the Rehai Geothermal Field (27 MAGs; distance: ~65 km) based on 16S rRNA gene identity with average nucleotide similarities greater than 99.4% (Fig. 4), which can be interpreted as members of the same species [84]. The polar amino acid ABC transporter was nearly universal in the genomes of aspartate primary utilizers (Supplemental Tables 4 and  6). Following aspartate transport, these genomes encoded enzymes capable of transferring carbon atoms from aspartate into pyrimidine nucleotides via multiple pathways, with the shortest being mediated by aspartate carbamoyltransferase (ATCase), which generates the pyrimidine biosynthesis pathway intermediate carbamoyl-aspartate directly from aspartate. Genes encoding the cation/acetate symporter, ActP, were less common in the genomes than polar amino acid transporters, but the protonated form, acetic acid, can freely diffuse across membranes [85]. Once inside the cell, acetate would need to be transformed into acetyl-CoA via acetate kinase-phosphate acetyltransferase (ACK-PTA) or acetyl-CoA synthetase (ACS) and then enter the TCA cycle via citrate synthase or converted to sugars via gluconeogenesis to generate precursors for nucleotide biosynthesis. However, there was no significant difference in the distribution or abundance of genes encoding ActP, ATCase, ACK-PTA, or ACS, or other enzymes directly involved in transformation of aspartate or acetate into nucleotides between primary utilizers and non-primary utilizers.

To screen more broadly for genotypes of primary utilizers versus non-primary utilizers, PCoA plots (Supplemental Figure 8) were generated based on gene content, and statistical tests were run to identify genes or KEGG modules that were significantly enriched or depleted in primary utilizers, but these analyses revealed no significant differences between primary utilizers and non-primary utilizers.

### High aspartate utilization may correspond to fast growth rate

**Fig. 5:**
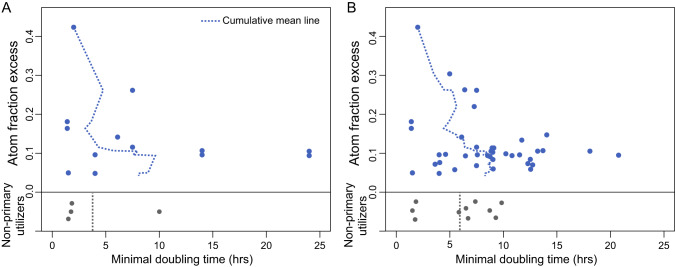
High aspartate utilization corresponds to fast maximum growth rate. Scatter plots showing aspartate AFE values and minimal doubling time based on literature (**A**) or literature and gRodon2 estimations (**B**). The dashed line represents the cumulative mean line.

Since gene content did not fully explain the preferred incorporation of carbon atoms from aspartate over acetate into DNA, we hypothesized that the broad utilization of aspartate incorporation might reflect differences in growth rate, since many aspartate-primary utilizers have smaller genomes and higher coding density compared to their counterparts (Supplemental Fig. 9A, B). Comparison of known laboratory growth rates (Fig. 5A; Supplemental Table 7) or growth rates predicted based on codon bias (Fig. 5B) revealed fast doubling times (<8 h) among the ASVs with the highest amounts of ^13^C incorporation from aspartate (>0.2 AFE). This pattern was not evident for acetate primary utilizers (Supplemental Fig. 10) and there was no difference between genome size or coding density between acetate primary utilizers and non-utilizers (Supplemental Fig. 11A, B).

## Discussion

### Sources of acetate and aspartate in geothermal springs

The sources and compositions of DOC in geothermal systems are complex [18]. Organic acids have been measured in high-temperature geothermal water in both acidic and circumneutral springs [21, 38] and in interstitial fluids of phototrophic mats, along with polar metabolites such as amino acids, sugars, and nucleotide bases [39]. Here, acetate and aspartate were chosen to represent two different components of the labile DOC pool to assess the organotrophic potential of a thermophilic microbial community. Acetate is a key metabolite for both intra- and intercellular metabolism and can be found universally in both oxic and anoxic environments, including geothermal springs [35–37]. Acetate could be produced biologically by microbes through fermentation or overflow metabolism during heterotrophic growth or by leakage of photosynthate by photoautotrophs [85]. Indeed, acetate is a major metabolite in hot spring chlorophototrophic mats [39, 86] that could supply DOC to microbes in hotter parts of spring systems, and acetogenesis from HCO_3_^-^ was detected in hot springs over a wide pH (3.5–8.5) and temperature range (60–80 °C) [87]. Thus, acetate could be both allochthonous or autochthonous in origin. Aspartate, a negatively charged amino acid, could be generated through cell lysis caused by viruses [88] or other mechanisms leading to cell lysis, such as parasitism or predation. But, unlike acetate, aspartate provides both carbon and nitrogen.

### The importance of aspartate for thermophiles

Our results showed higher bulk incorporation of aspartate over acetate into community DNA. Aspartate was also used more widely by the microbial community and was coupled to increases in the abundance of microorganisms in aspartate-amended microcosms and the specific taxa that incorporated it. Together, these data may suggest a broader importance of amino acid metabolism over organic acid metabolism in high-temperature systems. This result was somewhat surprising because acetate is a well-known carbon and energy source for prokaryotes [89] that can be readily converted to the fundamental metabolite acetyl-CoA via ACK-PTA or ACS, and because organic acids have been shown to be metabolized by cultivated thermophiles [90–93] and in situ in terrestrial geothermal springs [21, 22]. One possible explanation for the poor incorporation of acetate into DNA is that the acetate switch may have occurred because the initial concentration of acetate in our samples (~3.2 mM) was similar to the minimum acetate concentration known to induce the acetate switch (~1 mM) [94, 95]. However, this acetate concentration is lower than the concentrations that support the growth of many hyperthermophiles (6.1 to 12.2 mM) [90–93]. Another possible explanation is that acetate may have been assimilated broadly, but incorporated into other macromolecules, such as bacterial lipids. This caveat extends to all SIP approaches (e.g., DNA, RNA, lipids). While it is probable that different results would be obtained by examining other macromolecules, those would also suffer from biases according to the metabolism of each community member. Ultimately, the increase in 16S rRNA gene copy number and the strong positive correlation between aspartate incorporation into DNA and change in ASV abundance argue that there is indeed a community preference for aspartate in these experiments. Finally, it is possible that acetate amendment may have led to N starvation given that dissolved inorganic N concentrations are low in Gongxiaoshe Hot Spring [19, 20], so that acetate addition could result in the uncoupling of acetate assimilation from growth. On the contrary, aspartate has a C:N stoichiometry of 4:1, which is close to the Redfield ratio [96] and may be more favorable for growth of heterotrophic thermophiles. This interpretation is consistent with the heavy labeling of DNA from ammonia-oxidizing archaea (i.e., *Candidatus* Nitrosocaldus [97]) and nitrite-oxidizing bacteria (i.e., *Candidatus* Nitrotheca and *Candidatus* Nitrocaldera [98]) only in aspartate-amended incubations, which could reflect deamination of aspartate followed by oxidation of the resulting ammonia.

Aspartate is an important substrate for thermophiles. First, aspartate is the central metabolite in the aspartate metabolic pathway and is therefore a central metabolite for protein synthesis, nucleotide metabolism, tricarboxylic acid cycle, glycolysis, and other biosynthetic pathways [40, 41]. It can be converted into pyruvate and other intermediates in the TCA cycle, generating free energy for microbial growth. Additionally, aspartate is an important compatible solute to manage heat stress [99], and part of a large cytoplasmic pool of aspartate could flux into DNA synthesis [100]. However, it should be noted that our experiments do not rule out an important role for acetate, because acetate could be preferentially incorporated into other macromolecules such as bacterial lipids [101, 102]. ^13^C-acetate assimilation into lipids has previously been exploited to identify acetate utilizers in alkaline hot springs [38].

### Heterotrophy among thermophiles

Although high-temperature ecosystems are often discussed as ‘chemoautotrophic systems’ [103], our experiments show that many thermophiles readily assimilated aspartate and/or acetate. Some of the highly labeled taxa are well-known for their broad heterotrophic activities, such as *Thermus* [104], and members of the *Desulfurococcaceae*, including *Ignisphaera* [105, 106]. In contrast, although some members of the *Aquificae* were highly active assimilating both substrates, they are often thought of in the context of primary production. Yet, the assimilation of labile DOC, observed here in situ, is consistent with broad heterotrophic activity of some members of the *Aquificaceae* [107–109] and a member of the *Aquificaceae* isolated from nearby springs that uses acetate as an electron donor [93]. Other taxa that were highly labeled belong to uncultivated groups lacking known autotrophic pathways, such as *Candidatus* Fervidibacter [110] and *Candidatus* Kryptobacter [111]. The assimilation of aspartate by *Candidatus* Micrarchaeota is consistent with previous reports that some members of this phylum have the genomic capacity to utilize amino acids [112].

### Role of low-abundance taxa in geothermal systems and caution interpreting SIP data

The rare biosphere is often thought to be a transient or persistent “seed bank” of cells that is not fit to grow in a particular environment under the conditions in which it was sampled [113]. Yet, in our study, some low-abundance ASVs had the highest amounts of isotope incorporation, while the most abundant species had low isotope incorporation rates. This uncoupling of activity from cell abundance has been noted in other environments, such as the coastal marine pelagos, where low cell respiration rates for abundant *Pelagibacter* species were noted [114], despite other studies reporting high in situ rates of *Pelagibacter* growth, protein synthesis, and substrate assimilation [115]. The authors [114] suggested that the low respiration rate by *Pelagibacter* may not reflect low activity in general but instead the preferential use of rhodopsins for ATP synthesis over respiration. By extension, caution is warranted not to equate substrate incorporation into DNA in any stable isotope probing study to activity in general. For example, in our study, we measured high rates of incorporation of C atoms from aspartate into DNA of a low-abundance *Thermocrinis* ASV, but that same ASV did not incorporate C atoms from acetate into DNA. Some species of *Thermocrinis* use acetate as a carbon source [109], and ^13^C atoms from acetate have previously been traced into *Thermocrinis* lipids in situ in streamer communities [38]. Acetate incorporation into lipids is expected given the key role of acetate as a building block for acyl chains of bacterial lipids, but not archaeal lipids. Thus, we urge caution not to overinterpret low isotope incorporation because the choice of substrates may influence which taxa assimilate the substrate and how the substrate is processed in each species into macromolecules.

These caveats regarding low activity withstanding, the high activity of some members of the rare biosphere in our study is still remarkable. In our study, all of the microorganisms with high substrate assimilation rates (AFE > 0.2) were rare in the community at the time of sampling (<0.5%), and while some became more abundant during the corresponding incubation (*Micrarchaeales* (J92), *Ignisphaera* (J21), and *Acetothermiia* (J56)), others did not. Other studies have also noted increases in the abundance of some rare taxa in response to environmental perturbations [116], which can be interpreted in the context of relieving growth limitations. On the other hand, other studies have noted poor correlations between activity and abundance [117], which may be due to viral lysis, parasitism, or predation of the most active microorganisms. Indeed, many of the active taxa in our study belonged to the *Patescibacteria* and *Micrarchaeota*, which are typically inferred to be epibiontic symbionts of other prokaryotes, possibly acting as parasites or predators [118, 119], although some studies have challenged this broad interpretation [120]. Ultimately, our study reveals a complex relationship between activity and abundance that requires further study, particularly in geothermal systems.

## Conclusions

Carbon cycling in hydrothermal systems has rarely been explored in situ. In this study, we applied qSIP to investigate the utilization of distinct pools of labile DOC, acetate and aspartate, by thermophiles in Gongxiaoshe Hot Spring. The microbial community responded to both substrates, although only aspartate was coupled to net increases in abundance, and most taxa preferentially incorporated aspartate over acetate into DNA, possibly reflecting the dynamic cycling of cell lysis products within the spring ecosystem (e.g., due to viral lysis) or the favorable C:N stoichiometry of aspartate over acetate. We also leveraged rich metagenomic datasets from these springs, which allowed us to go beyond typical qSIP studies that focus on 16S rRNA gene variants to look into phylogenetic and genomic determinants for the utilization of the different labile DOC pools, revealing a correlation between high aspartate assimilation and fast growth rate. The broad utilization of aspartate was congruent with the near ubiquity of the polar amino acid ABC transport system. Fast growth rate and membrane transporters for organic compounds may endow some members of thermophilic communities, including rare taxa, to respond quickly to influxes of organic matter during monsoons or other ecosystem perturbations. Further studies could probe the fates of a more diverse suite of organic compounds to determine whether observed resource partitioning extends to other substrates and whether specialist/generalist paradigms reported in other ecosystems extend to simplified microbial communities that inhabit extreme environments.

### Supplementary information

Supplemental Figures

Supplemental Tables

## Data Availability

MAGs matched to ASVs are available at NCBI (BioProject: PRJNA974368). Raw reads associated with qSIP experiment have been uploaded to the NCBI Sequence Read Archive (BioProject: PRJNA981310). All other data are included as supplemental material to this paper.
