# Supplementary material for: Resource partitioning and amino acid assimilation in a terrestrial geothermal spring
Source: ISME J. 2023 Sep 23;17(11):2112–22. doi: 10.1038/s41396-023-01517-7 (PMC10579274; doi:10.1038/s41396-023-01517-7)
Supplement: Supplementary file 1 — Supplemental Figures [file 41396_2023_1517_MOESM1_ESM.pdf]

Resource partitioning and amino acid assimilation in a terrestrial geothermal spring

Dengxun Lai, Brian P. Hedlund, Rebecca L. Mau, Jian-Yu Jiao, Junhui Li, Michaela Hayer, Paul Dijkstra, Egbert Schwartz, Wen-Jun Li, Hailiang Dong, Marike Palmer, Jeremy A. Dodsworth, En-Min Zhou, Bruce A. Hungate

Supplementary Tables

1. **Table S1**. ASV profiles in fractions of samples
2. **Table S2.** Taxonomy, atom fraction excess and genome information
3. **Table S3.** The relative abundance of ASVs in initial samples and atom fraction excess
4. **Table S4.** List of genes involved in substrate assimilation and metabolism into DNA for aspartate and acetate utilizers
5. **Table S5.** Full list of genome annotation by Eggnog-mapper
6. **Table S6.** BlastP results for aspartate and acetate transporters
7. **Table S7.** Aspartate utilization status
8. **Table S8.** Acetate utilization status

Supplementary Figures

1. **Figure S1.** Linear discriminative analysis effect size (LEfSe) of microbial abundance at multiple taxonomic levels with 13C-acetate and 13C-aspartate incubations
2. **Figure S2.** Effect of incubation on alpha diversity
3. **Figure S3.** Aspartate enhances the growth of some taxa
4. **Figure S4.** Distribution of atom fraction excess values among ASVs
5. **Figure S5.** Aspartate utilization shows a positive phylogenetic autocorrelation across short phylogenetic distances (low taxonomic rank)
6. **Figure S6.** High activity of rare taxa
7. **Figure S7.** Metabolic pathways showing the incorporation of aspartate and acetate into nucleotides
8. **Figure S8.** Similar gene content in acetate primary utilizers and non-primary utilizers and aspartate primary utilizers and non-primary utilizers
9. **Figure S9.** Smaller genome and higher coding density in genomes of aspartate-primary utilizers
10. **Figure S10.** Acetate utilization does not correspond to maximum growth rate
11. **Figure S11.** No significant differences in genome size, coding density, and GC content in genomes of acetate-primary utilizers

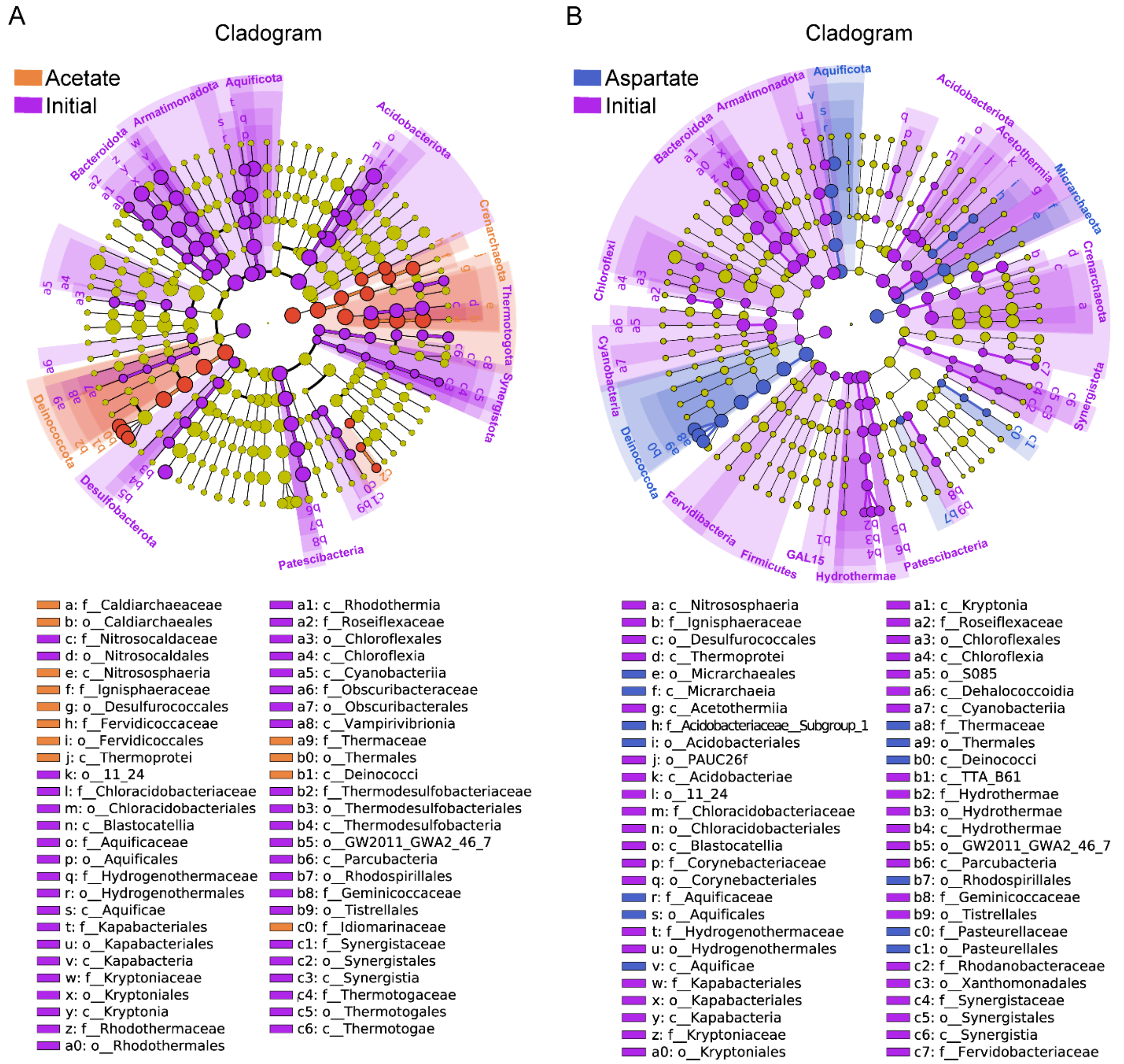

Figure S1. Linear discriminative analysis effect size (LEfSe) of microbial abundance at multiple taxonomic levels with ^{13}C-acetate and ^{13}C-aspartate incubations. (A) Cladogram represents the taxa that were more abundant in the initial samples (purple) and samples incubated with ^{13}C-acetate (orange). Yellow nodes represent taxa with no significant change in abundance during incubations. (B) Cladogram represents the taxa that were more abundant in the initial samples (purple) and samples incubated with ^{13}C-aspartate (blue).

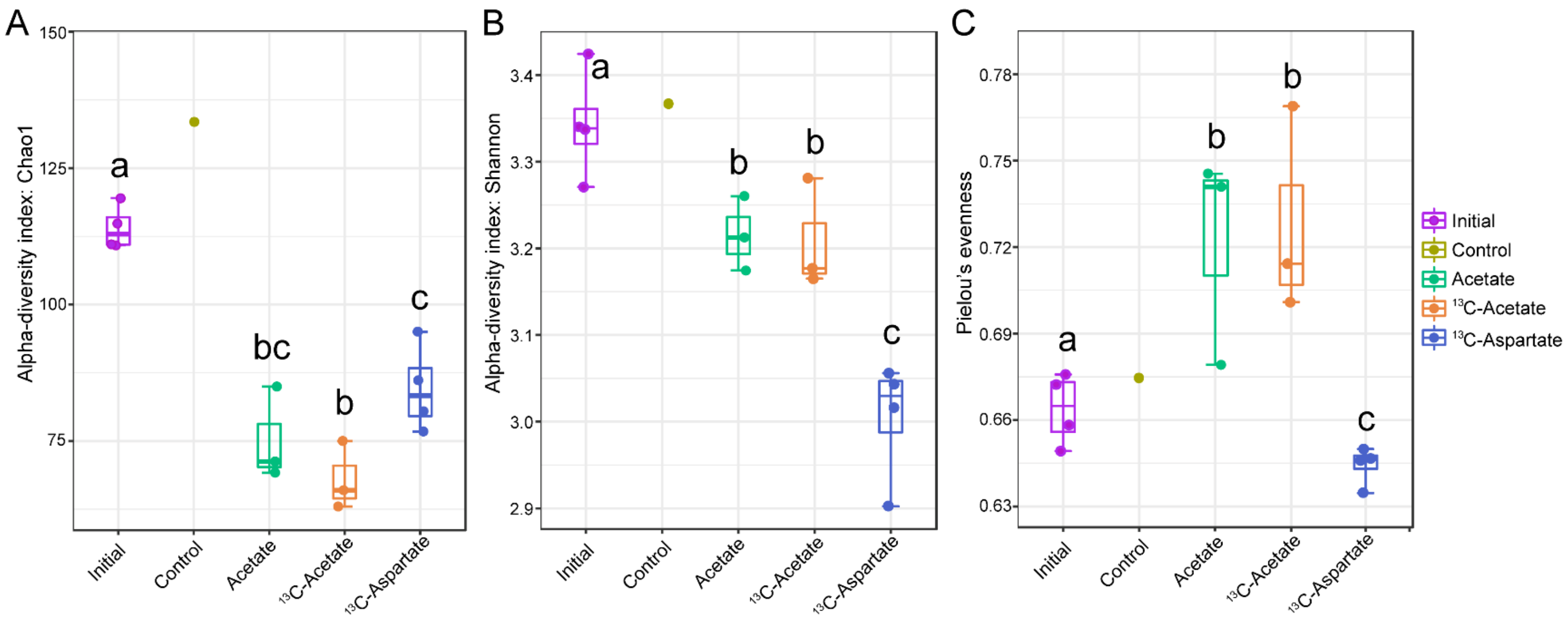

Figure S2. Effect of incubation on alpha diversity. Microbial alpha diversity as assessed with the Chao1 index (A), Shannon diversity index (B) and Pielou's Evenness (C) in the five groups: initial (purple), control (brown), natural abundance acetate (green), ^{13}C-acetate (orange), and ^{13}C-aspartate (blue). The same lowercase letter indicates no significant difference based on Welch's t-test ($p > 0.05$). The analysis shows that incubation with any substrate significantly reduced both richness and Shannon diversity (versus initial).

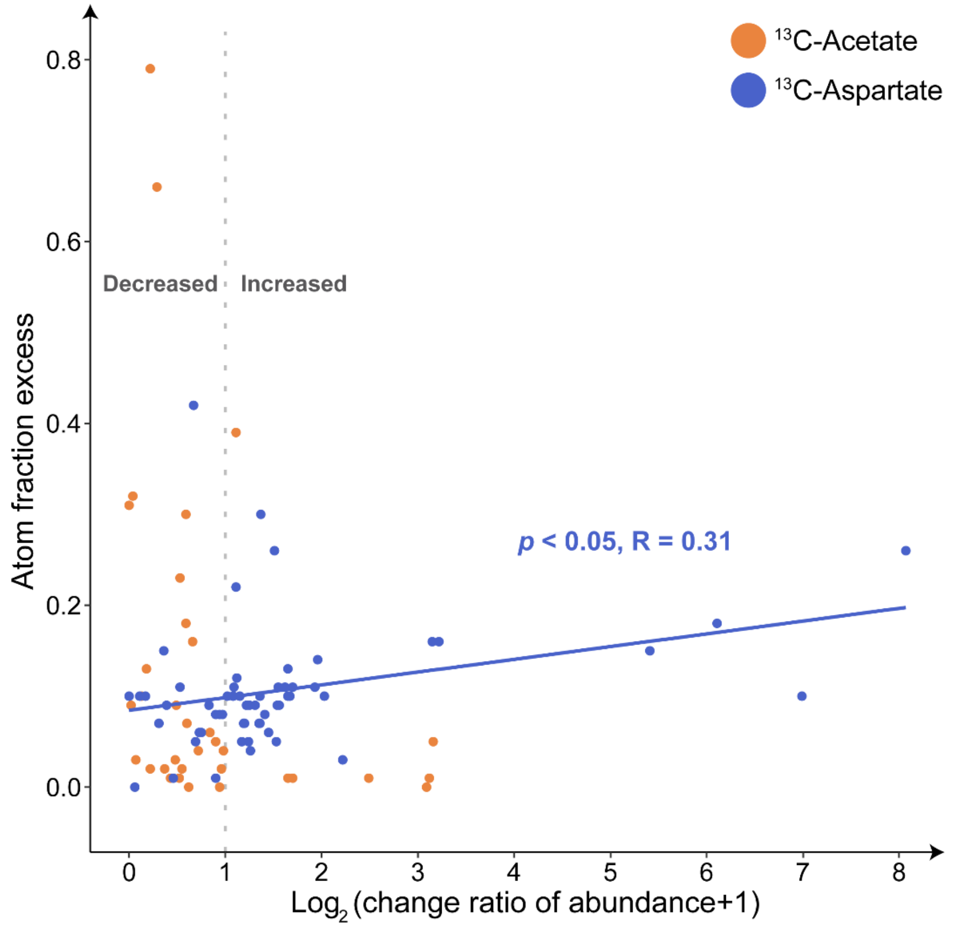

Figure S3. Aspartate enhances the growth of some taxa. Association between the change ratio of absolute abundance and AFE of ^{13}C-acetate (orange) and ^{13}C-aspartate (blue). The statistical significance of the relationships was based on Pearson correlation coefficient test.

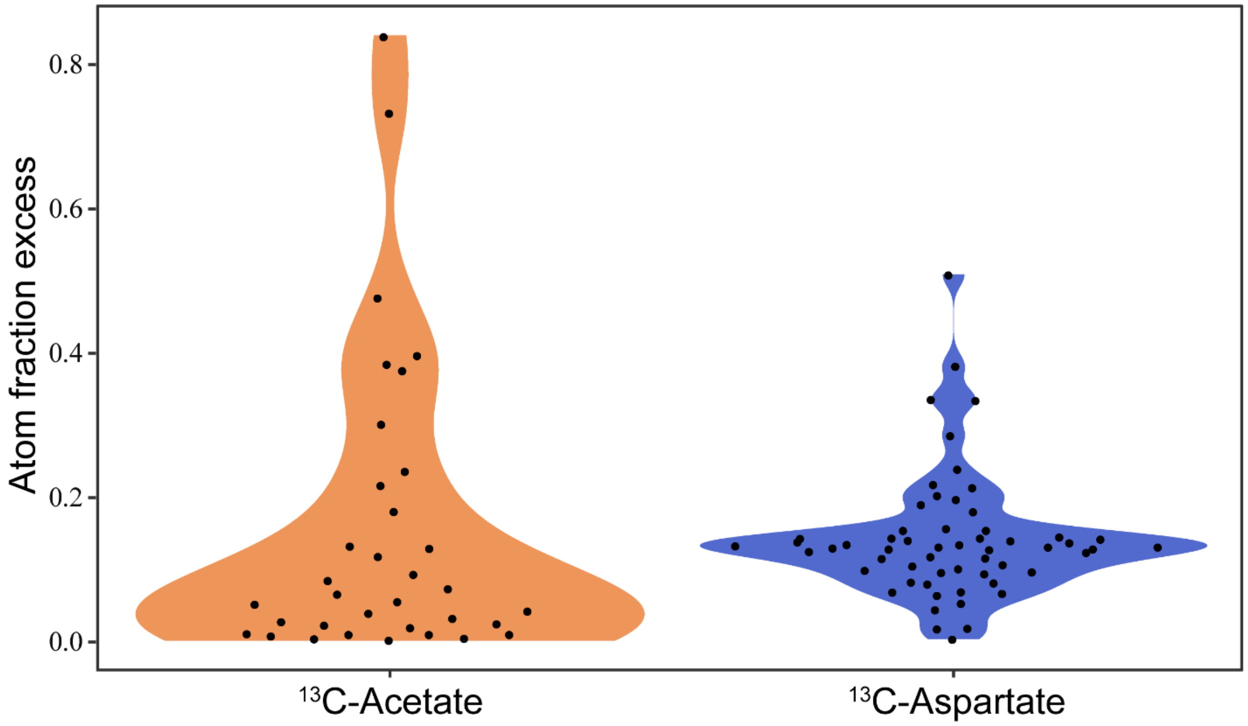

Figure S4. Distribution of atom fraction excess values among ASVs. ASVs enriched with ^{13}C-acetate (orange) or ^{13}C-aspartate (blue), which are not significantly different (Asymptotic General Independence Test, $p = 0.499$).

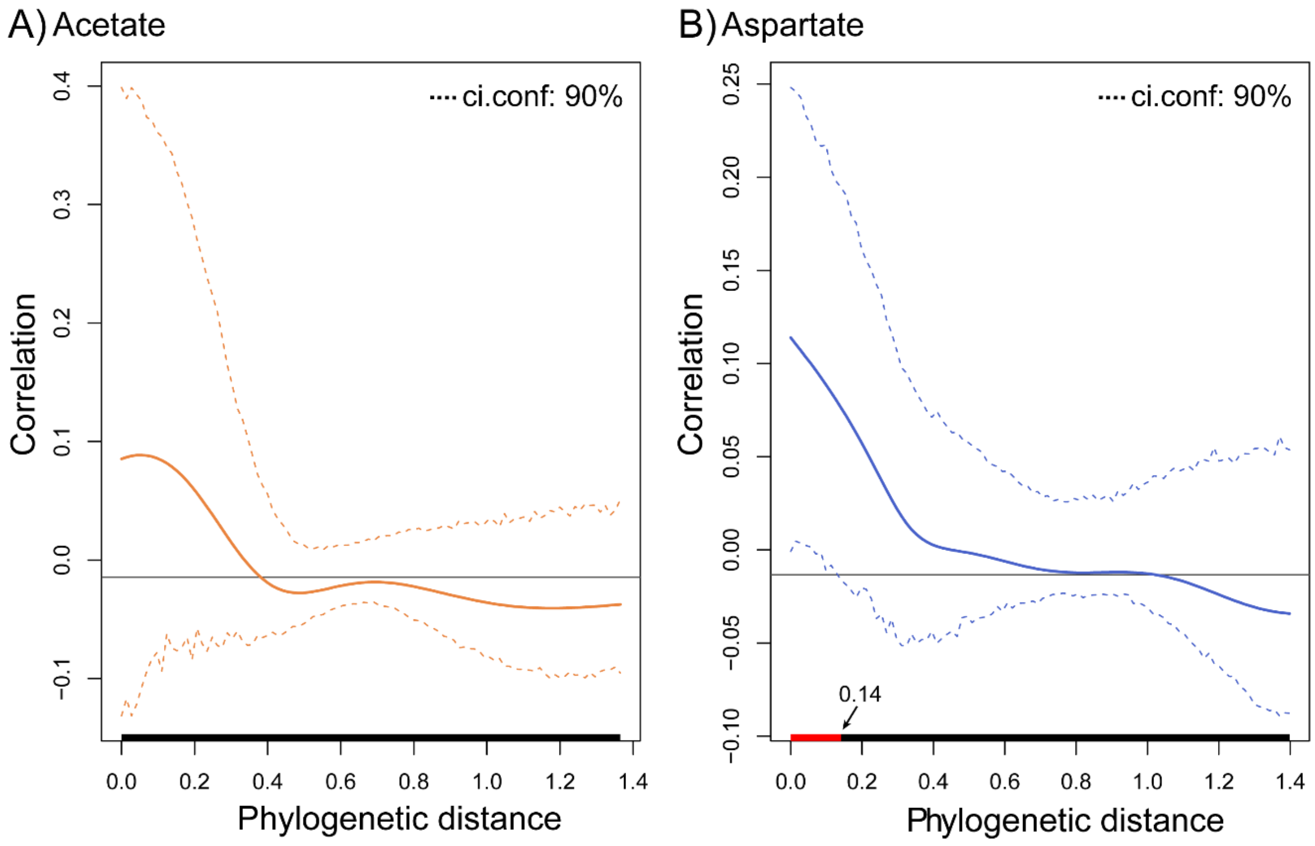

Figure S5. Aspartate utilization shows a positive phylogenetic autocorrelation across short phylogenetic distances (low taxonomic rank). Phylogenetic correlograms for acetate (orange) and aspartate (blue) datasets. Moran's I autocorrelation is represented by the solid line, and the lower and upper bounds of the 90% confidence interval, which was computed using nonparametric bootstrap resampling are shown by the dashed lines. The horizontal black line represents the expected Moran's I values under the null hypothesis of no phylogenetic autocorrelation. Red shown on the x-axis indicates a significant positive autocorrelation and black indicates no autocorrelation. There was no significant autocorrelation above 0.14 phylogenetic distance for aspartate utilization.

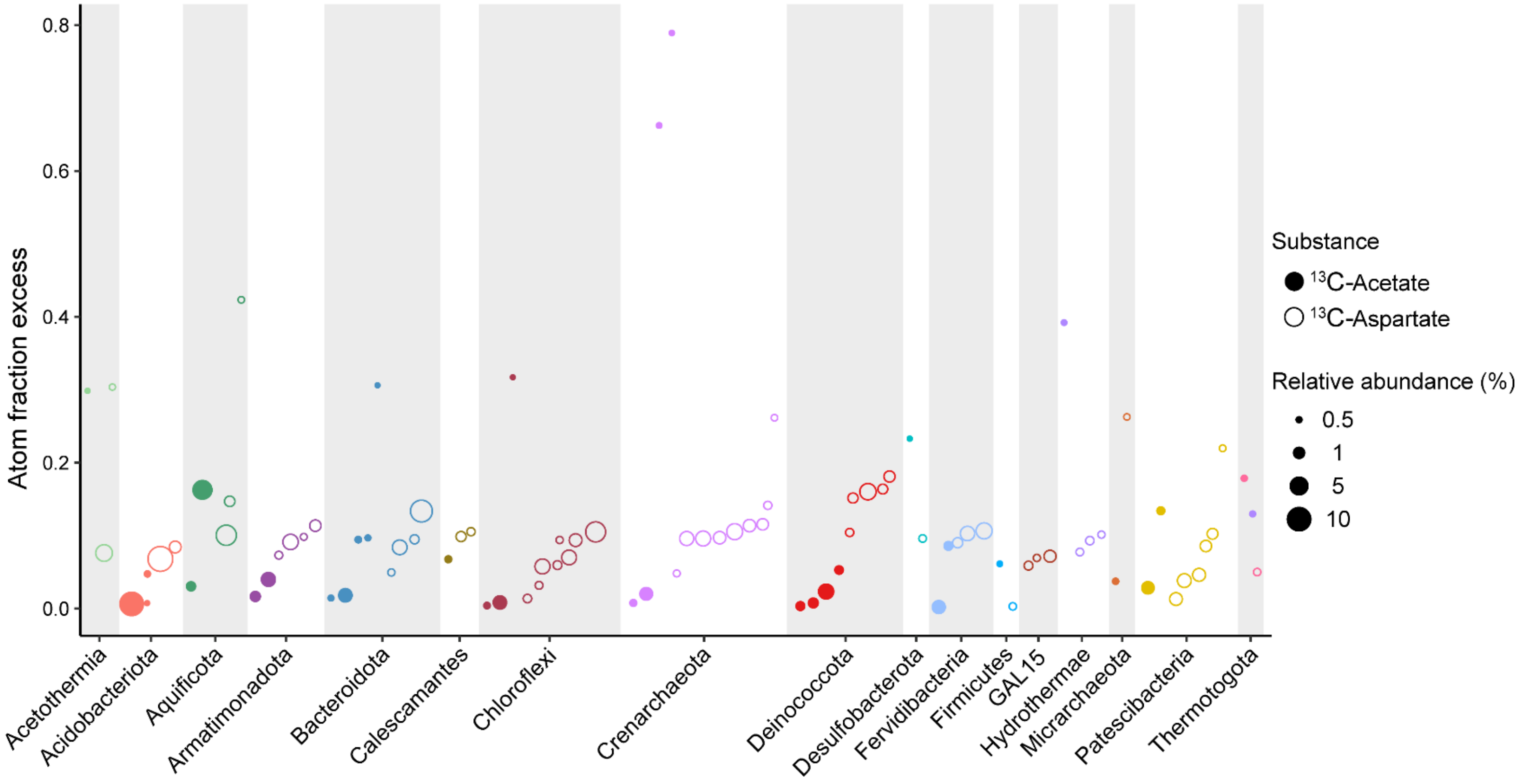

Figure S6. High activity of rare taxa. Scatter plot showing atom fraction excess of ^{13}C after incubation with ^{13}C-acetate (solid circle) or ^{13}C-aspartate (hollow circle) in ASVs belonging to different phyla. Dot size represents average relative abundance in initial samples (time 0). For both substrates, the most active taxa are rare (<0.5 %). Refer to Supplemental Table 3 for detailed information about ASVs used in the figure.

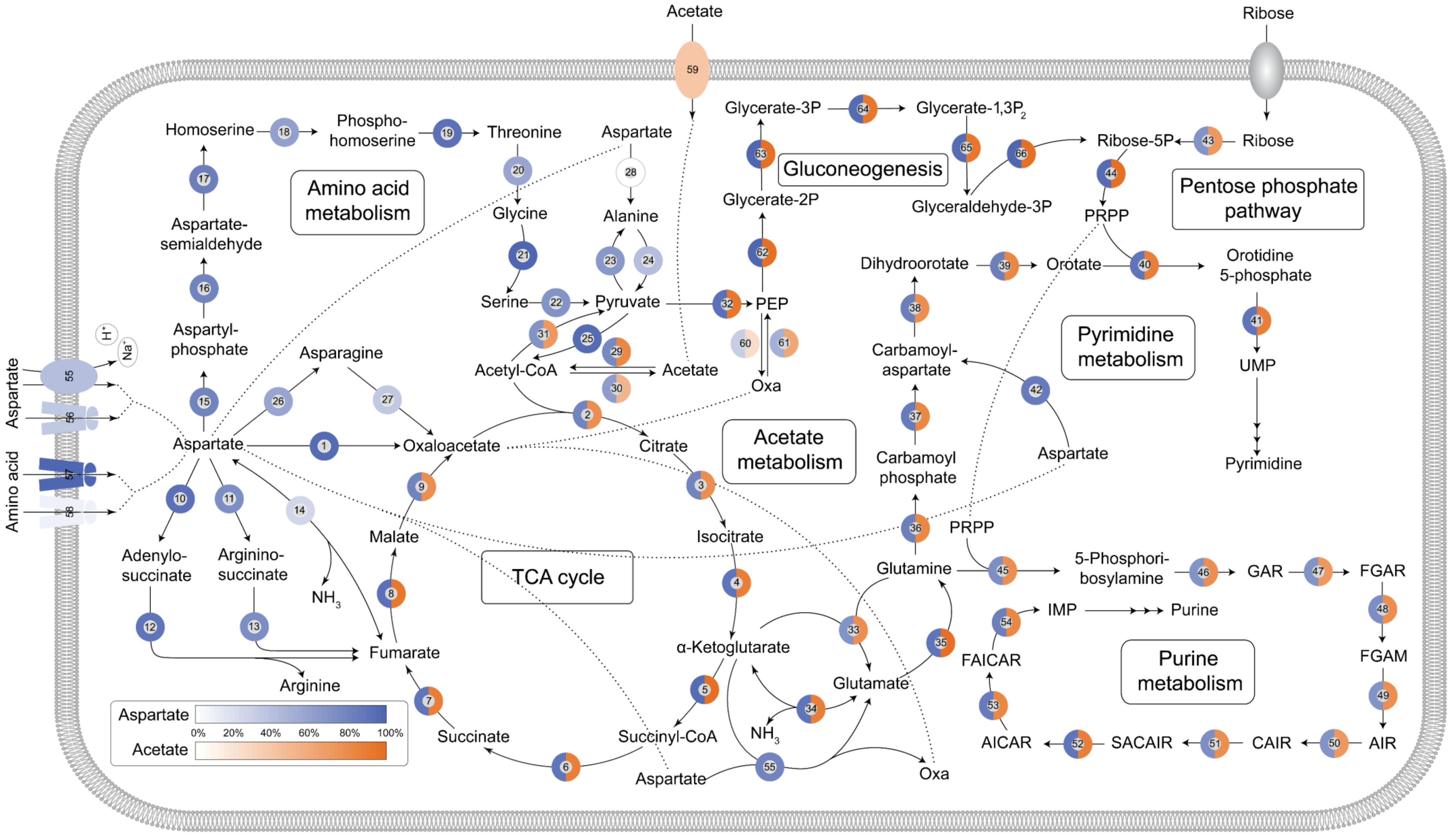
Acetate
Ribose
Amino acid metabolism
Gluconeogenesis
Pentose phosphate pathway
Pyrimidine metabolism
Acetate metabolism
TCA cycle
Purine metabolism
Homoserine
Phospho-homoserine
Threonine
Aspartate
Glycine
Alanine
Serine
Pyruvate
Acetyl-CoA
Glycerate-3P
Glycerate-1,3P2
Glycerate-2P
Glyceraldehyde-3P
Ribose-5P
PRPP
PEP
Oxa
Aspartate-semialdehyde
Aspartyl-phosphate
Asparagine
Oxaloacetate
Citrate
Isocitrate
α-Ketoglutarate
Succinyl-CoA
Succinate
Fumarate
Malate
Adenylo-succinate
Arginino-succinate
Arginine
NH3
Dihydroorotate
Orotate
Orotidine 5-phosphate
UMP
Pyrimidine
Carbamoyl-aspartate
Carbamoyl phosphate
Glutamine
Glutamate
5-Phosphori-bosylamine
GAR
FGAR
FGAM
AIR
CAIR
SACAIR
AICAR
FAICAR
IMP
Purine
H+
Na+
Aspartate
Amino acid
Aspartate
Acetate
0% 20% 40% 60% 80% 100%

Figure S7. Metabolic pathways showing the incorporation of aspartate and acetate into nucleotides. 32 high-quality genomes (>90% completeness and <5% contamination) matched to the ASVs showing aspartate utilization and 19 high-quality genomes matched to ASVs showing acetate utilization were screened. The shades of color represent the proportion of genes detected in the genomes. The map indicates the incorporation of aspartate and acetate into nucleotides is feasible.

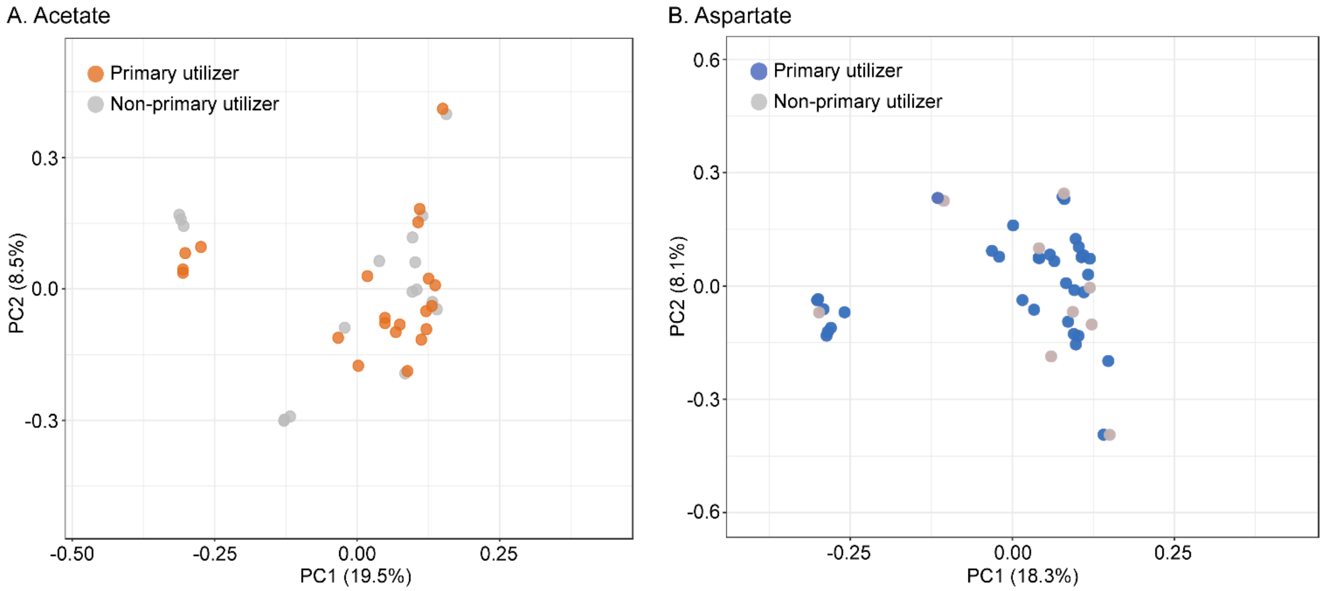

Figure S8. Similar gene content in (A) acetate primary utilizers and non-primary utilizers and (B) aspartate primary utilizers and non-primary utilizers. Principal coordinates analysis (PCoA) plot with Bray-Curtis dissimilarity based on the functional profiling of genomes annotated by KEGG (Supplemental table 4).

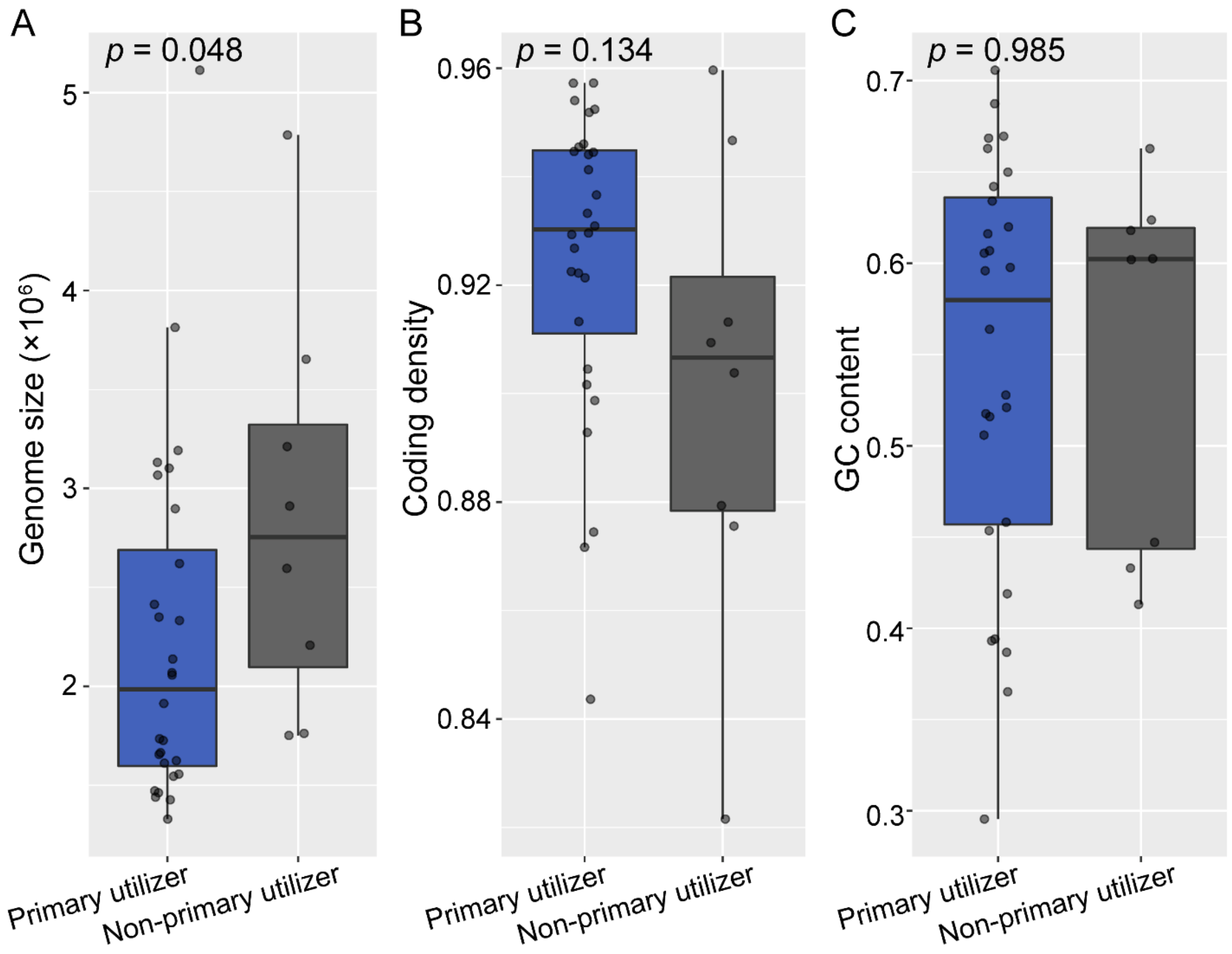

Figure S9. Smaller genome and higher coding density in genomes of aspartate-primary utilizers. Boxplot comparison of (A) genome size, (B) coding density and (C) GC content between aspartate-primary utilizers (blue) and non-primary utilizers (grey). Statistical significance was measured by Wilcoxon exact rank sum test.

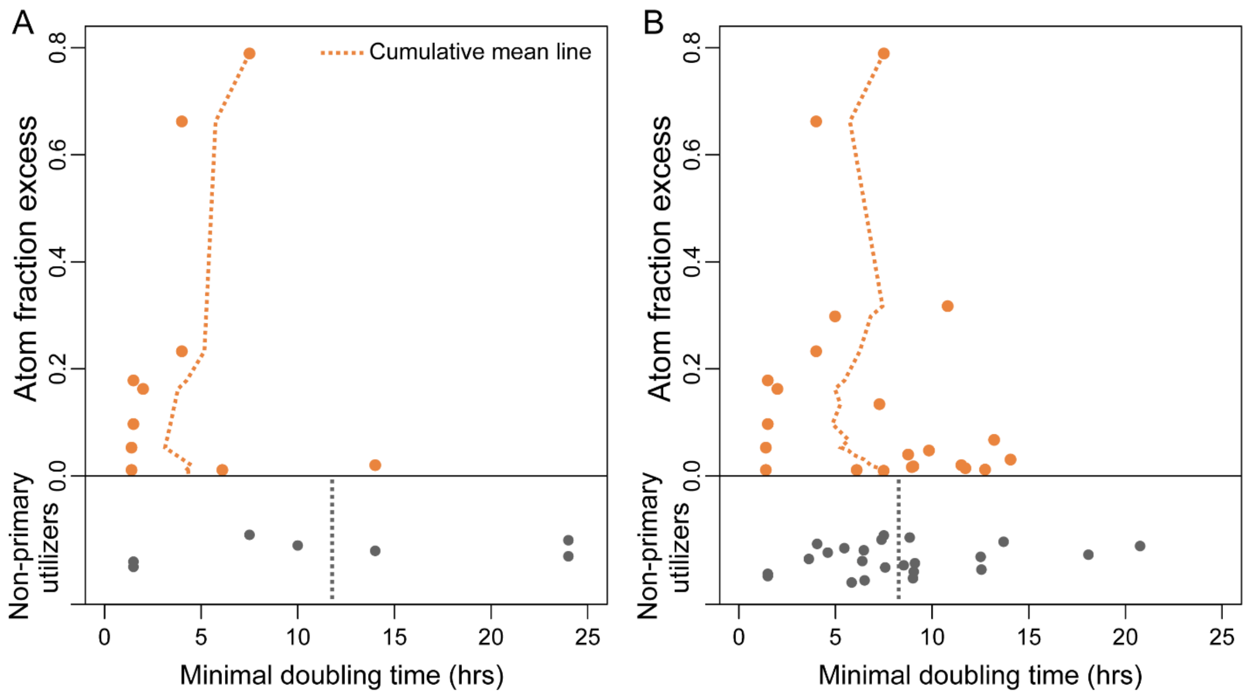

Figure S10. Acetate utilization does not correspond to maximum growth rate. Scatter plots showing the acetate AFE values and minimal doubling time (A) based on literature or (B) literature and gRodon2 estimations. The dashed line represents the cumulative mean line.

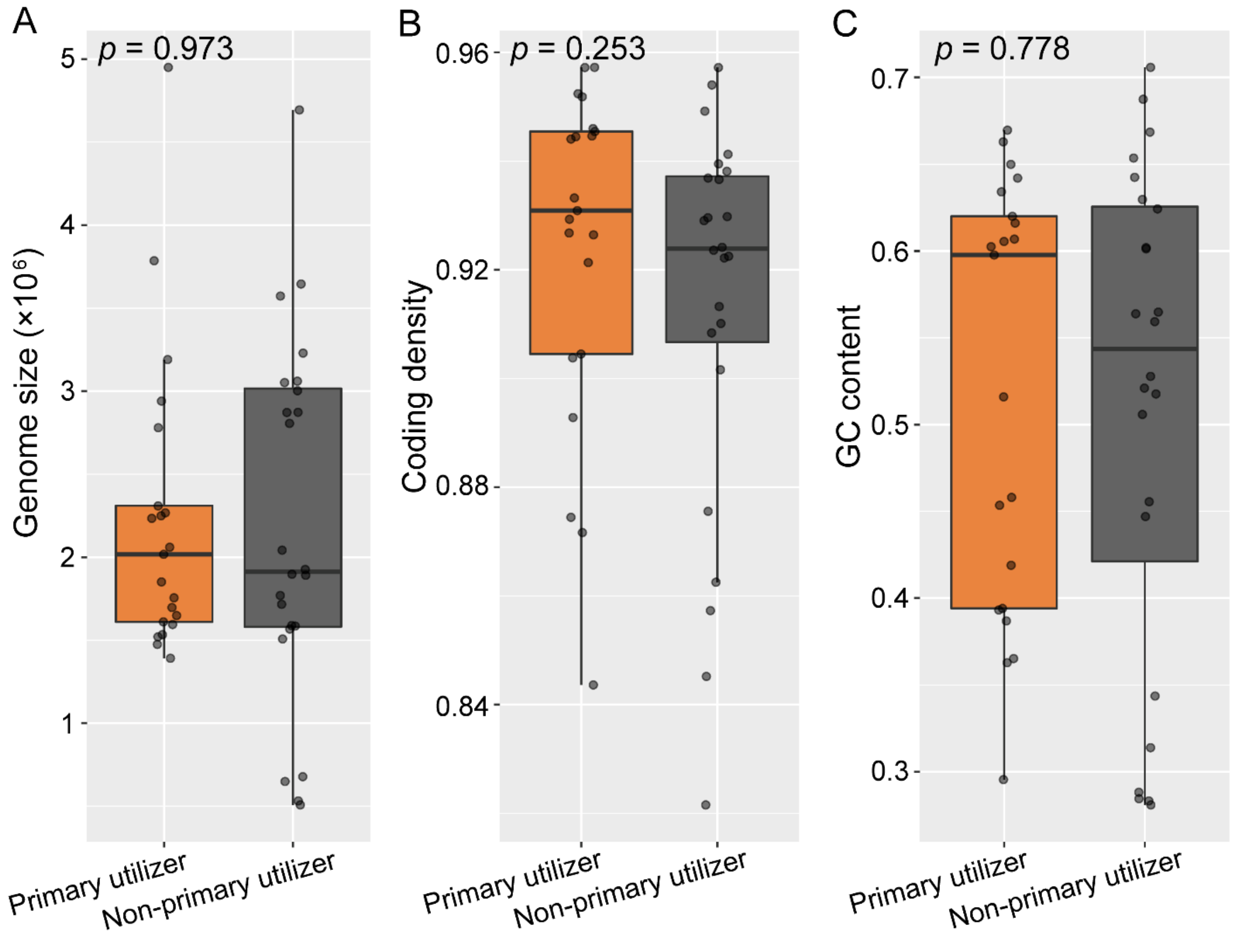

Figure S11. No significant differences in genome size, coding density, and GC content in genomes of acetate-primary utilizers. Boxplot comparison of (A) genome size, (B) coding density, and (C) GC content between acetate-primary utilizers (blue) and non-primary utilizers (grey). Statistical significance was measured by Wilcoxon exact rank sum test.
